# A data set of inland lake catchment boundaries for the Qiangtang Plateau

**DOI:** 10.1038/s41597-019-0066-x

**Published:** 2019-05-16

**Authors:** Denghua Yan, Meng Li, Wuxia Bi, Baisha Weng, Tianling Qin, Jianwei Wang, Pierre Do

**Affiliations:** 1 State Key Laboratory of Simulation and Regulation of Water Cycle in River Basin, China Institute of Water Resources and Hydropower Research (IWHR), Beijing, 100038 China; 2 Institute of Water Resources and Hydrology Department of Hydraulic Engineering, Tsinghua University, Beijing, 100084 China; 3 College of Hydrology and Water Resources, Hohai University, Nanjing, 210098 China

**Keywords:** Limnology, Hydrology

## Abstract

A catchment is the basic unit for studying hydrologic cycle processes and associated climate change impacts. Accurate catchment delineation is essential in the field of hydrology, environment, and meteorology. Traditionally, catchment delineation is most easily carried out where the outflow area can be easily determined because of a well-defined outlet. The obstacle of the current study is to determine accurately the catchment boundary of lakes that are internally draining and, therefore, lack a well-defined outflow (i.e. inland lakes). This study describes a catchment delineation method which demarcated all the catchments of the lakes in the Qiangtang Plateau, especially for the inland lakes and their closed catchments. Lake catchment boundaries determined for the Qiangtang Plateau provide a significant advancement for water resource and climate change evaluation and agriculture production in the area.

## Abstract (summary)

**Table:** 

Design Type(s)	modeling and simulation objective • data transformation objective • image processing objective
Measurement Type(s)	drainage basin
Technology Type(s)	computational modeling technique
Factor Type(s)	Classification
Sample Characteristic(s)	Tibetan Plateau • lake

Machine-accessible metadata file describing the reported data (ISA-Tab format)

## Background & Summary

The Qiangtang Plateau, located on the Qinghai-Tibet Plateau^1^ which has high concentration of glaciers^2^ and permafrost^3^, contains the most inland lakes in the world^4^. Because of the current context of climate change evidenced by rising temperature^5^, this region displays an acceleration of warming^6^, glaciers melting^7^, an intensification of hydrological processes, development of alluvial fans, and significant expansion of lakes^8^. The Qiangtang Plateau has a unique geography marked by high elevation, extended lakes and mountains, sparse vegetation, geological fragmentation and shallow soil with very little anthropogenic influence due in part to a sparse population. These features make it a key area for global research on climate change^9^ because these changes can be studied in isolation of other anthropogenic factors.

Lakes, the most important aquatic component of the Qinghai-Tibet Plateau, are especially widely spread inside the plateau^10^. Their variations (formation and disappearance, expansion and contraction) are significantly sensitive to climate change. Lakes in the Qiangtang Plateau are not only a reflection of the water cycle^11^, but also the ecological barrier in order to maintain the ecological resilience of the Qiangtang Plateau. Under global warming, lake areas have changed substantially. On the one hand, due to the retreat and melting of glaciers, the flow rate has increased^12^ and lake areas have expanded; on the other hand, due to the increase of evaporation^13^, lake areas have shrunk or even disappeared. There have been mixed responses of lakes to climate change. The change of lake area will further affect the plateau grassland, which is directly related to the development of local agriculture and animal husbandry, and the survival of herders. Meanwhile, the inland lakes and their surface hydrologic connections could influence multiple ecological processes^14^. Therefore, it is of great significance to explore the evolution of lakes and their water balance, also the lake connectivity^15^. The determination of lake catchments is the basis for establishing a hydrological model and calculating the water balance relationship of lakes.

At present, catchments can be divided into two types: with outlets and without outlets (inflow basin). Catchments of lakes that have obvious outlets can relatively easily be determined, thus there are many relevant study results at present. At present, the automatic watershed segmentation method based on Digital Elevation Models (DEM) is usually applied to lakes with outlets. However, for catchments without obvious outlets, there are few studies, thus this paper focused on solving this problem. We targeted the inland lakes on the Qiangtang Plateau since most inland lakes on the Qinghai-Tibet Plateau are located at the Qiangtang Plateau and the observational data is seriously lacking.

Meanwhile, in these last decades, researchers extracted, from field investigations and remote sensing, lake boundaries to study these changes, and produced distribution maps of the Qinghai-Tibet Plateau lakes^16,17^. However, these studies have considered lakes as the main unit of study and not catchments as the basic unit. Moreover, current research is limited to several large lake basins^18–20^ and do not include all the lakes in the Qiangtang Plateau.

This study mainly contains three parts: (i) extracting all lakes of the Qiangtang Plateau and calculating their area separately; (ii) producing the detailed classification of their network positions: single lake, tandem lakes, and mixed lakes; (iii) determining the catchments of the above three types of lakes respectively. In producing this catchment set, we used a combination of manual and programming method to achieve semi-automated catchment extraction. The acquisition of lake area in the Qiangtang Plateau can provide support for the study of lake evolution law. The acquisition of lake catchments can provide direct support for the establishment of hydrological models, understanding the water balance relationship of lakes, and conducting water resources evaluation. In addition, these results can give some indirect support for climate change response and impact on agricultural production.

## Methods

Lake catchment extraction relied on several calculation processes based on DEM, such as depression filling, flow direction, flow accumulation, basin outlet selection, basin generation, and other processes^21,22^. The dataset used the ASTER GDEM V2 (publicly available on ‘https://search.earthdata.nasa.gov/’) with a spatial resolution of 30 m and developed jointly by the Japanese METI and the US NASA.

Identifying a DEM’s flow outlet and filling local depressions are essential to properly delineate lake basins^23^. We used this method to delineate lake catchments in open drainage basins, i.e., those with an outlet that flows to the edge of the DEM. However, this method may not be appropriate for endorheic basins, as these basins do not discharge to a river or ocean. For these lakes, a catchment delimitation should consider the outlet as flowing inside the basin. For example, on the Qiangtiang Plateau, basically all the rivers drain inward toward lakes, representing the inner outlets.

We can determine each catchment boundary based on the location of the lake. Thus, we should extract lake water body in the Qiangtang Plateau first. The method is described below.

### Lake water body extraction

Water bodies, given their absorbency, reflect a specific wavelength detected by satellites. This characteristic allows them to be distinguished from other objects on the landscape. However, other objects like mountain shadows disturb the radiation emission because their band reflectivity is similar to that of water bodies. To more clearly distinguish lakes, we compared the intensity of reflections associated to water molecules to weaker signal or ‘noise’ in order to maximize water wavelengths and discriminate them. This extraction process uses the ratio method model, also called NDWI (Normalized Difference Water Index)^24^ method. The satellites images come from Landsat 8 OLI (Operational Land Imager).

Mcfeeters (1996) defined the NDWI or normalized water difference index (Eq. (1)) as:

1 $$NDWI=\frac{{\rho }_{Green}-{\rho }_{NIR}}{{\rho }_{Green}+{\rho }_{NIR}}$$

Where ${\rho }_{Green}$ stands for the green band of the remote sensing image, the third band in the Landsat 8 OLI image; ${\rho }_{NIR}$ stands for the near infrared band of the remote sensing image and the fifth band in the Landsat 8 OLI image.

After calculating the NDWI value, we extracted lakes from the resulting raster layer by applying a threshold to these values. In general, we found that lake extractions were better when the NDWI is higher than zero. But for more accurate water body extraction, we adjusted the threshold based on visual interpretation (i.e. comparing the extracted lakes with remote sensing images) and Google Earth. In this way, the extracted water body better reflects reality. Under these circumstances, the lake extractions were best when the NDWI is higher than 0.15. This NDWI threshold may vary when applied outside the Qiangtang Plateau.

**Fig. 1:**
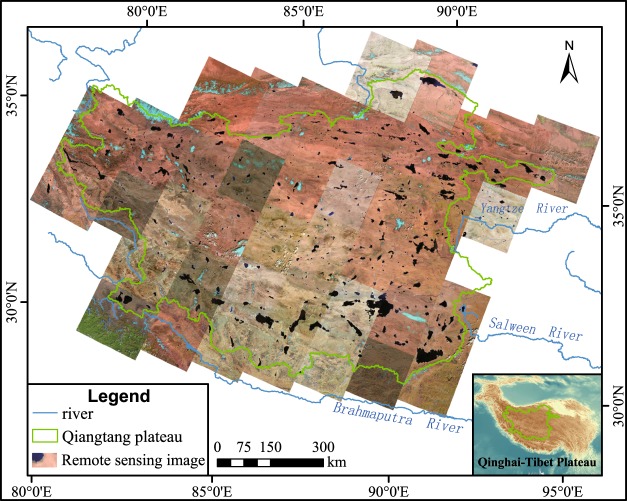
Remote sensing image of Landsat 8 in the Qiangtang Plateau.

**Fig. 2:**
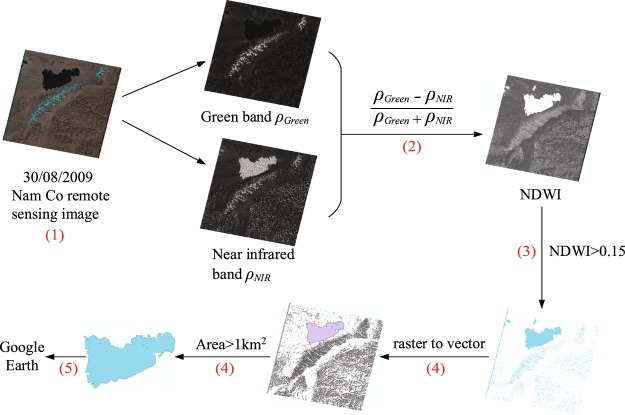
Water body extraction processes (e.g. Nam Co).

To ensure the extraction accuracy of the lakeshore, we manually selected cloudless remote sensing images. It required 41 Landsat 8 images of the Qiangtang Plateau to cover the study area (Fig. 1). The remote sensing images provided the green and near-infrared bands for the NDWI calculations. After classification and renumbering process, programming extracted water bodies in bulk. We used a Python (2.7.5) script to execute ArcGIS (10.2) commands to complete the remote sensing image calculation, screening and raster vectorization, eventually forming the range of water body. Specific extraction processes are in Fig. 2. The details are as follows:

1. We selected the remote sensing images of the green and near-infrared wave bands from the downloaded images in different periods from 1970s to 2010s. Then numbered them following “ID_Green” and “ID_NIR” respectively, where ID was the number of each image, and numbered from 1 to the last scene image.
2. Process the green and the near-infrared wave band of each scene according to the numbering cycle, and the NDWI value was calculated with Eq. 1.
3. Select NDWI threshold 0.15 as the critical value that best distinguishes between water and other features, extracting the grids of NDWI > 0.15.
4. Convert extracted lakes from raster to vector graphics area. Select lakes with an area greater than 1 km^2^. As the statistics of lakes in Bulletin of First National Census for Water in China concern the lakes with an area greater than 1 km^2^, the area threshold of the selection was defined as 1 km^2^. In addition, the area threshold cannot influence the determination of lake catchment boundaries.
5. Convert lake vector graphics files into KMZ files and import into Google Earth. Remove the clutter noise of non-water parts (such as glaciers, etc.) based on the visual paths.

### Lake catchment delineation

**Fig. 3:**
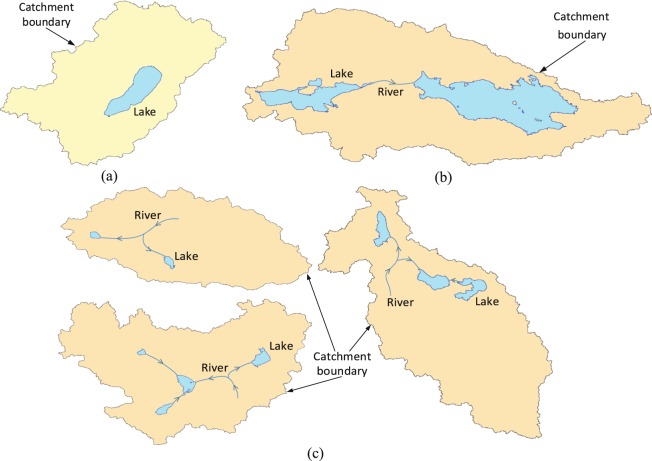
Diagram of different lake and lake catchments in the Qiangtang Plateau. (**a**) Single-lake catchment. (**b**) Tandem-lake catchment. (**c**) Mixed-lake catchment.

This study focused on the lakes without apparent outlets. Before delineating the lake catchments, it is necessary to first check whether there are hydrologic connections among the lakes. If there are hydrologic connections, the lakes with hydrologic connections should be divided into a lake catchment. Based on the analysis of relationships among the lakes in the Qiangtang Plateau, three main relationships can be found in the Qiangtang Plateau: single lake, tandem lake and mixed lake (Fig. 3). Single lake refers to a single lake that has no hydrologic connection with the other lakes (Fig. 3a). Tandem lake refers to multiple lakes in the same basin which have upstream and downstream hydrologic relationships (Fig. 3b). Mixed lake refers to several lakes originating from the same glacial river, if they have obvious relationship of mainstream and tributaries, there is a larger river to replenish one of the lakes and a smaller river to replenish another lake (Fig. 3c). The methods for obtaining the lake catchments of these three types of lakes are not exactly the same, thus we described the methods of obtaining the lake catchments for these three lakes types, respectively.

### Single-lake catchment

Single-lake catchment refers to a lake that has its own separate catchment area, with no hydrologic connection to the other lakes surrounding it (Fig. 3a). In the Qiangtang Plateau, such lake is of relatively simple type, and its catchment division steps are as follows:

1. Determine the probable catchment area by visually examining with the mountains and rivers in Google Earth, and sketch out the initial peripheral boundary of the lake catchment. The boundary should be slightly expanding to the outside of the ridge line. Then export the file in KMZ format (Fig. 4a).

**Fig. 4:**
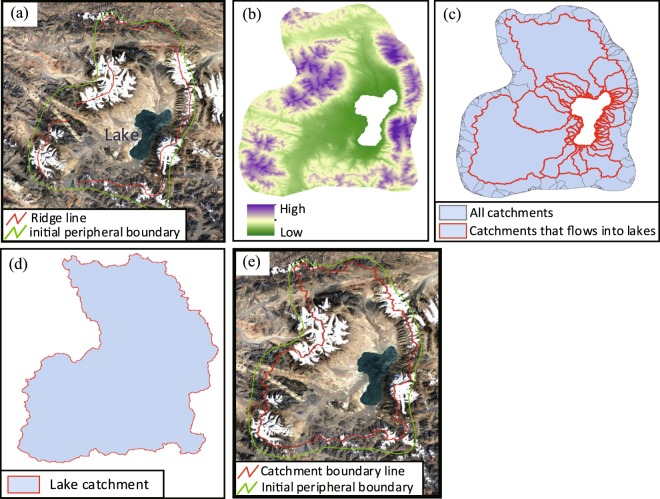
Single-lake catchment division processes. (**a**) Outline initial boundary. (**b**) Extract and erase DEM. (**c**) Generate and select catchments. (**d**) Get the catchment boundary. (**e**) Match and confirmation.
2. Import the KMZ file from step (1) into ArcGIS software to form a vector surface pattern, which is used to cut the DEM.
3. Remove the elevation data in the lake area from the cut DEM, obtaining the DEM data to be used to delineate the lake catchment (Fig. 4b). The lake is removed to create an outlet for flow for the internally-draining basin.
4. Use the ArcGIS hydrologic tools (Fill, Flow Direction, Flow Accumulation, and Basin) on the DEM obtained from step (3) to condition and prepare the DEM for delineation. This step results in a set of basins that flow to the internally-draining lake (Fig. 4c).
5. Combine with the Flow Accumulation and select all the catchment units flowing into the lake from the generated basins in step (4). Dissolve the border between these catchments and fill the lake vacancies (when there are some “holes” inside the generated catchment) to obtain the lake catchment (Fig. 4d).
6. Export the lake catchment obtained in step (5) into KMZ file and then import into Google Earth for comparison (Fig. 4e). If the boundary is unreasonable (the catchment boundary should be within the initial boundary and the rivers are flowing into the lake), adjust the initial boundaries, and repeat the processes above.

### Tandem-lake catchment

The tandem-lake catchment refers to those with two or more lakes with upstream and downstream hydrologic connections that should be included in one catchment when doing some studies (Fig. 3b). Its catchment delineation procedure is as follows:

1. Same as step (1) for the single-lake catchment delineation (Fig. 5a).

**Fig. 5:**
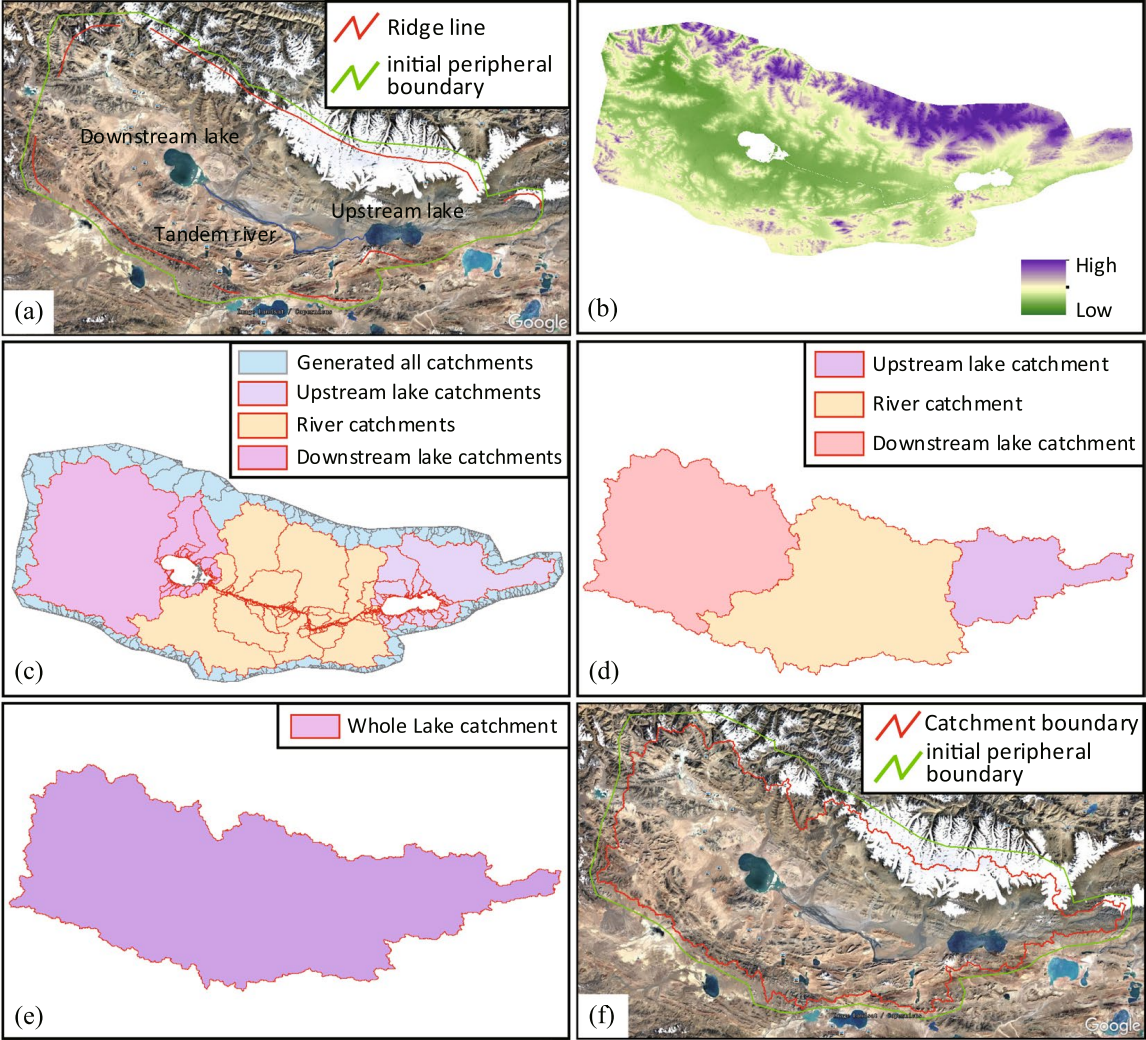
Tandem-lake catchment division processes. (**a**) Outline initial boundary. (**b**) Extract and erase DEM. (**c**) Generate and select catchments. (**d**) Get the lake and river catchment boundary. (**e**) Get the whole catchment boundary. (**f**) Match and confirmation.
2. In Google Earth, manually delineate rivers that link lakes (the rivers and lakes can be easily identified in Google Earth). Export the rivers as a KMZ file (Fig. 5a).
3. Import the KMZ file from steps (1) and (2) into ArcGIS and convert both to vector files. Make the river a buffer with a width 60 m. Then extract the DEM by the initial peripheral boundary.
4. Remove the pixel data in the lake area and the river buffer from the DEM obtained from step (3) to obtain the final DEM to be used to delineate the catchment (Fig. 5b).
5. Use the ArcGIS hydrologic tools (Fill, Flow Direction, Flow Accumulation, and Basin) on the DEM obtained from step (4) to condition and prepare the DEM for delineation (Fig. 5c).
6. Combine with the Flow Accumulation and select all the catchment units flowing into the lake and river from the generated catchment in step (5). Dissolve the border between these catchments and fill the lake vacancies to obtain the lake catchment. Merge all catchments (Fig. 5d) to obtain the whole tandem-lake catchment (Fig. 5e).
7. Export the lake catchment obtained in step (6) into KMZ file and then import into Google Earth for comparison (Fig. 5f). If the boundary is unreasonable (the catchment boundary should be within the initial boundary and the rivers are flowing into the lake), adjust the initial boundaries, and repeat the processes above.

### Mixed-lake catchment

In addition to single-lake catchment and tandem-lake catchment, the remaining lake catchment is classified as a mixed-lake catchment. It refers to two or more lakes with much more complex hydrologic links, such as those flowing from the same river into different lakes or rivers of two lakes eventually merging into the same lake, etc. (Fig. 3c). These lakes should be taken into consideration by one catchment. Its catchment delineation procedure is as follows:

1. Same as step (1) for the single-lake catchment delineation (Fig. 6a).

**Fig. 6:**
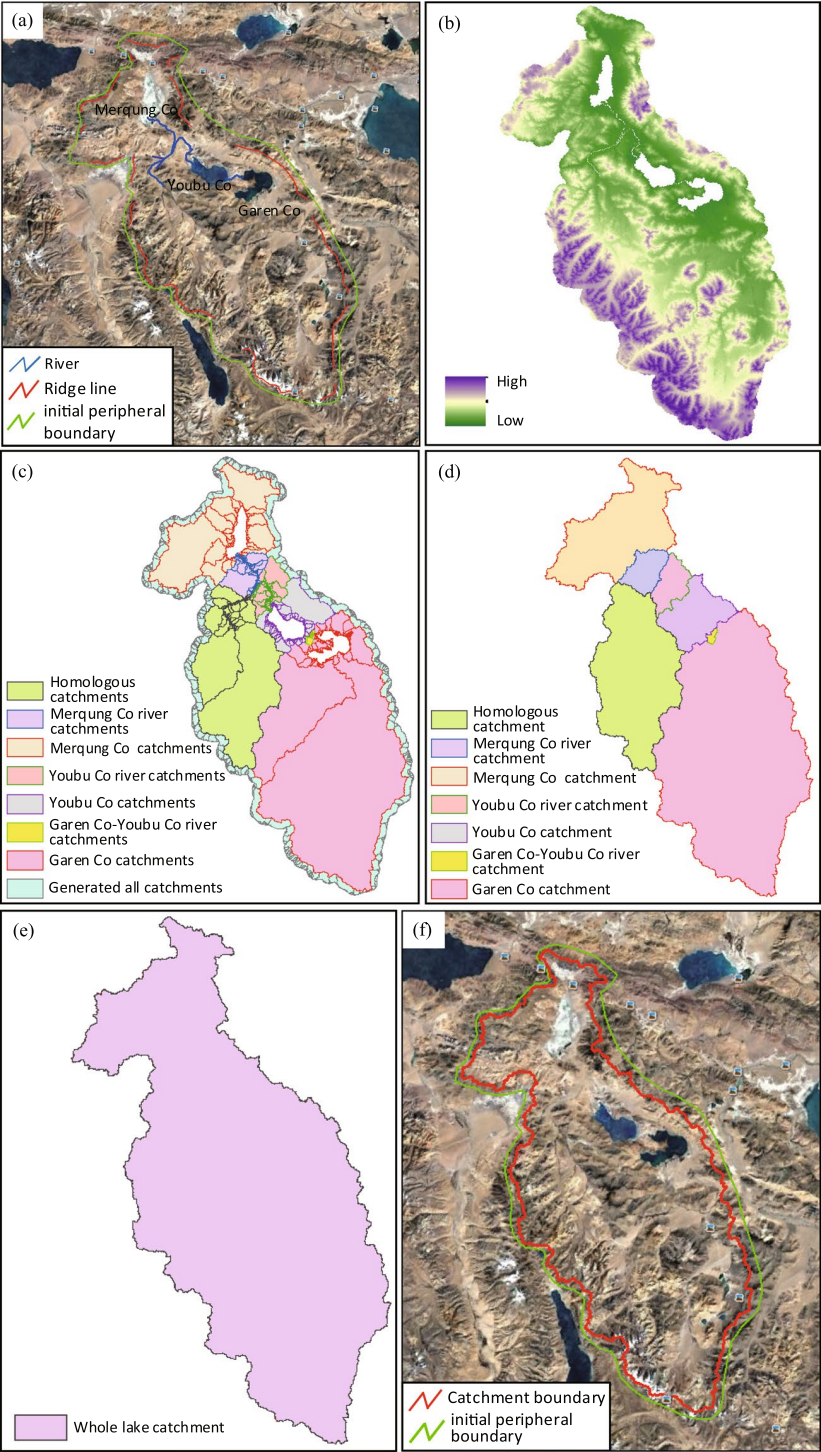
Mixed-lake catchment division processes. (**a**) Outline initial boundary. (**b**) Extract and erase DEM. (**c**) Generate and select catchments. (**d**) Get the lake and river catchment boundary. (**e**) Get the whole catchment boundary. (**f**) Match and confirmation.
2. In Google Earth, manually delineate rivers that link lakes. Export the rivers as a KMZ file (Fig. 6a).
3. Import the KMZ file from steps (1) and (2) into ArcGIS and convert both to vector files. Make the river a buffer with a width 60 m. Then extract the DEM by the initial peripheral boundary.
4. Remove the pixel data in the lake area and the river buffer from the DEM obtained from step (3) to obtain the final DEM to be used to delineate the catchment (Fig. 6b).
5. Use the ArcGIS hydrologic tools (Fill, Flow Direction, Flow Accumulation, and Basin) on the DEM obtained from step (4) to condition and prepare the DEM for delineation (Fig. 6c).
6. Combine with the Flow Accumulation and select all the catchment units flowing into the lake, mainstream and tributary from the generated basins in step (5). Dissolve the border between these catchments and fill the lake vacancies to obtain the lake catchment. Merge all catchments (Fig. 6d) to obtain the whole mixed-lake catchment (Fig. 6e).
7. Export the lake catchment obtained in step (6) into KMZ file and then import into Google Earth for comparison (Fig. 6f). If the boundary is unreasonable (the catchment boundary should be within the initial boundary and the rivers are flowing into the lake), adjust the initial boundaries, and repeat the processes above.

The delineation of the mixed-lake catchment is similar to that of the tandem. The vital step to delineate mixed-lake catchments is to extract the DEM to include the full lake and river extents so that the generated catchment area has the outlet. The difference is that due to the relatively complex hydrologic linkage among different lakes, there is not a simple upstream and downstream relationship in the mixed-lake catchment, but also other intertwined relationships such as cognate lakes. Therefore, when delineating the mixed-lake catchment, the shared catchments of different lakes should be divided to facilitate the discussion of their spatial distribution of water quantity.

Although there are some differences between the tandem lakes and mixed lakes, the steps are similar. Therefore, the first two steps and the last two steps need to be manually operated, while the other steps can be programmed using the Python platform to simplify duplication and to improve efficiency.

## Data Records

**Fig. 7:**
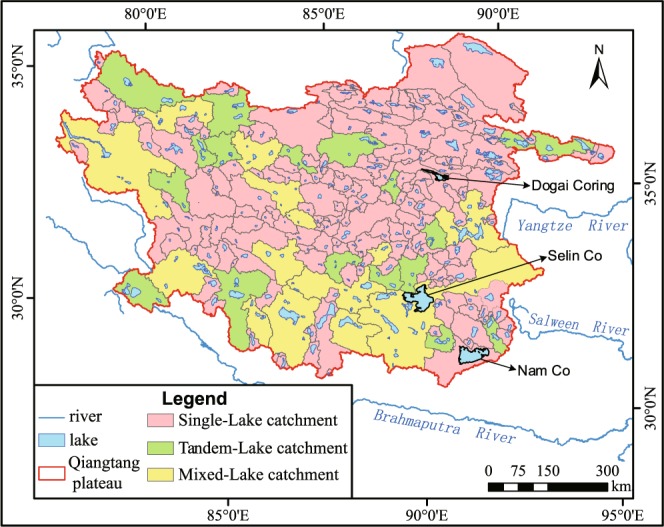
Lake catchment distribution map of the Qiangtang Plateau.

A total of 225 catchments were delineated with our process. Among them, 193 are single-lake catchments with a total area of 398,163 km^2^, accounting for 57.1% of the total area of the Qiangtang Plateau (698,000 km^2^); 18 are tandem-lake catchments, with a total area of 107,739 km^2^, accounting for 15.4%; 14 are mixed-lake catchments, with a total area of 192,147 km^2^, accounting for 27.5% (Fig. 7).

The data set is available within Figshare^25^, and is in four folders. The first folder, “Catchment”, contains 3 shapefiles: single-lake catchment, tandem-lake catchment, and mixed-lake catchment. The second folder, “Lake”, contains a shapefile with all lakes derived from Landsat 8 remote sensing images. The lake distribution map was the basis of the lake catchment delineation process. The shapefiles in both folders used an Albers isometric projection. The third folder, “Information”, contains an excel file which is used to explain the affiliation between lakes and catchments. The fourth folder, “Python Scripts”, contains two python scripts which are mentioned in the section “Lake catchment delineation”. The data set is open access^25^.

Table 1 shows data labels and descriptions of the “Lake” shapefile. Some small lakes may have no names, so we named them by their nearby lakes with an orientation. For example, “Xin Lake_east” represents a lake close to the east of Xin Lake.

**Table 1:** Data labels and descriptions for the “Lake” shapefile.

Data label	Description
SHAPE	Feature type of the lake object
Lake_ID	Lake code
Catch_ID	The catchment code to which the lake belongs
NAME_CN	Chinese name of the lake
NAME_EN	English name of the lake
LAT_NORTH	North latitude of the geometric center of the lake polygon in decimal degree
LON_EAST	East longitude of the geometric center of the lake polygon in decimal degree
PERI_km	Perimeter of the lake polygon (km)
AREA_km^2^	Area of the lake polygon (km^2^)

Table 2 shows data labels and descriptions of the three “Catchment” shapefiles in detail. “Catch_ID” represents the code of each catchment and “Lake_ID” is the unique identifier of each lake within each catchment. The “Lake_ID” corresponds to the label of “Lake.shp”. The single-lake catchment is named after the name of the lake in it (i.e., columns Ca_NAME_CN and Ca_NAME_EN). The tandem-lake catchment is named after the lake where the water finally converges. And the mixed-lake catchment is named after the largest lake where the water finally converges.

**Table 2:** Data labels and descriptions for the “Catchment” shapefile.

Data label	Description
SHAPE	Feature type of the catchment object
Catch_ID	Catchment code
Ca_NAME_CN	Chinese name of the catchment
Ca_NAME_EN	English name of the catchment
LAT_NORTH	North latitude of the geometric center of the catchment polygon in decimal degree
LON_EAST	East longitude of the geometric center of the catchment polygon in decimal degree
PERI_km	Perimeter of the catchment polygon (km)
AREA_km^2^	Area of the catchment polygon (km^2^)
Lake_ID	The code of lakes in the catchment
La_NAME_CN	Chinese name of the lake in the catchment
La_NAME_EN	English name of the lake in the catchment

## Technical Validation

During the data set development, location and name of the lakes were verified based on the “Encyclopedia of Rivers and Lakes in China”^26^. After lake extraction, they were compared to all the lakes from the literature to ensure the accuracy of the lake extraction and the reliability of the name source.

After delineating the catchments, we took the three biggest catchments and compared them with the literature to determine whether the boundaries of the catchment are accurately located on the ridge line around the lake. If the boundary line was very different from the ridge line, it was modified and delineated along the ridge line. For the tandem-lake and mixed-lake catchments, the connecting rivers in them were carefully drawn according to the Google Earth. This hand drawing also helped to verify internal hydrologic linkages among lakes. Combined with the DEM, we could use the elevation to determine the upstream and downstream lakes. Finally, we scanned pages with the catchments of only a few larger lakes in the “Encyclopedia of Rivers and Lakes in China” and corrected the location of the scanned lake through the extracted lake. We then compared the catchment boundary in our data set with that in the book to verify the validity of the data set. A lot of time and energy were spent to carefully check each catchment and got the final catchment data set.

**Fig. 8:**
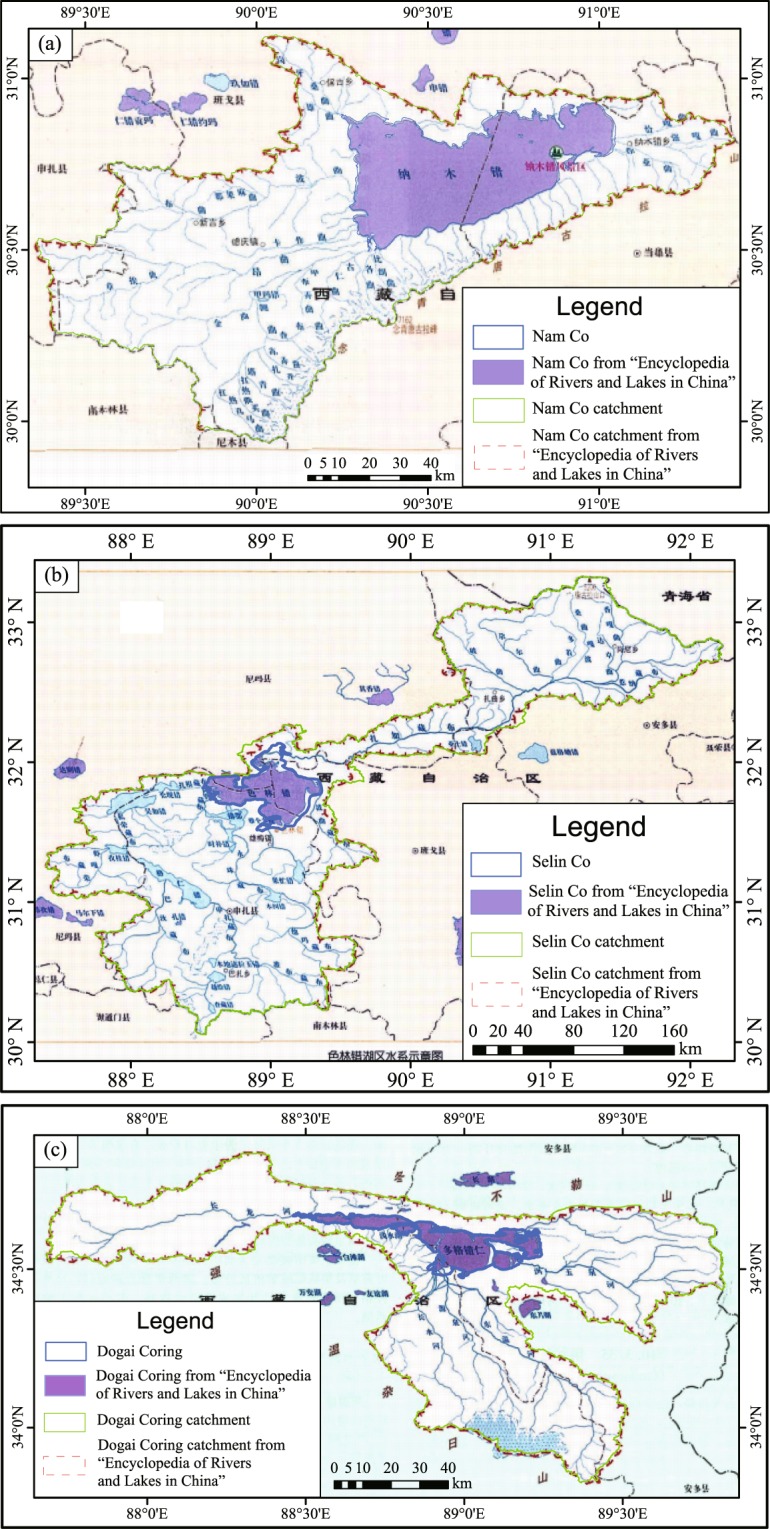
Comparison of the selected catchment boundaries in our data set and that in the “Encyclopedia of Rivers and Lakes in China”. (**a**) Nam Co Catchment. (**b**) Selin Co Catchment. (**c**) Dogai Coring Catchment.

There are only three lakes that were given their catchment boundaries in “Encyclopedia of Rivers and Lakes in China”. Thus, we scanned them and located the scanned picture to compare with our data set. Figure 8a–c were the comparison of Nam Co, Selin Co and Dogai Coring catchment boundaries in our data set and that in the “Encyclopedia of Rivers and Lakes in China”. It can be seen that the boundaries of our data set are consistent with that of the scanned map from the book. For example, the lake catchments area of Nam Co is 10,610 km^2^ in the “Encyclopedia of Rivers and Lakes in China”, while of 10,731.32 km^2^ in our data set. In addition, the lake and catchments areas generated by our data set are similar to which in the previous studies. For the Nam Co Lake, the lake area mentioned in previous studies is about 2018 km^2^ and 2028.50 km^2^ in 2010^19^ and 2014^16^, respectively; while the lake area in our data set is 2018.55 km^2^ in 2010s.

## ISA-Tab metadata file

Download metadata file

## Data Availability

As mentioned above, some catchment delineation steps can be programmed using Python scripts which was provided in our data set. It contains two scripts, one is for single-lake catchment delineation named “Single-lake catchment.py” and the other is for tandem-lake catchment and mixed-lake catchment delineation named “Tandem-lake and Mixed-lake catchment.py”. Before running these two scripts, it needs to install ArcGIS 10.1 or above version and install the python function carried by it in the Windows system. Then, the scripts can be opened by the IDLE (python GUI). As for “Single-lake catchment.py”, line 9 is for setting a workspace for program running. It should put the initial peripheral boundary shapefile named “bd.shp” and lake shapefile named “lake.shp” in the workspace folder. Line 10 is for the original DEM grid file setting. By finishing all the above steps, it will automatically generates all the catchment units named “basin.shp”. As for “Tandem-lake and Mixed-lake catchment.py”, add the “river.kmz” file which is the river depicted from Google Earth into the workspace folder, the rest setting is the same as the “Single-lake catchment.py”.
