# Author Correction: A data set of inland lake catchment boundaries for the Qiangtang Plateau

**DOI:** 10.1038/s41597-019-0176-5

**Published:** 2019-09-06

**Authors:** Denghua Yan, Meng Li, Wuxia Bi, Baisha Weng, Tianling Qin, Jianwei Wang, Pierre Do

**Affiliations:** 1 State Key Laboratory of Simulation and Regulation of Water Cycle in River Basin, China Institute of Water Resources and Hydropower Research (IWHR), Beijing, 100038 China; 2 Institute of Water Resources and Hydrology Department of Hydraulic Engineering, Tsinghua University, Beijing, 100084 China; 3 College of Hydrology and Water Resources, Hohai University, Nanjing, 210098 China

Correction to: *Scientific Data* 10.1038/s41597-019-0066-x, published online 16 May 2019

Following publication of this Data Descriptor, it was found that the Normalized Difference Water Index (NDWI) threshold was incorrectly stated in the text. In all instances where the NDWI threshold is stated as 1.5 the actual value used was 0.15.

In addition to the above, it was found that the blue overlay used in Figure 1 to indicate the presence of a river incorrectly intersected the inset image in the lower right.

These errors have been corrected in both the HTML and PDF versions of this Data Descriptor.

